# Elevated Inorganic Carbon Concentrating Mechanism Confers Tolerance to High Light in an Arctic *Chlorella* sp. ArM0029B

**DOI:** 10.3389/fpls.2018.00590

**Published:** 2018-05-07

**Authors:** Kwon Hwangbo, Jong-Min Lim, Seok-Won Jeong, Jayaraman Vikramathithan, Youn-Il Park, Won-Joong Jeong

**Affiliations:** ^1^Plant Systems Engineering Research Center, Korea Research Institute of Bioscience and Biotechnology, Daejeon, South Korea; ^2^Department of Biological Sciences, Chungnam National University, Daejeon, South Korea

**Keywords:** CCM, high light tolerance, ROS, photodamage, *Chlorella*

## Abstract

Microalgae and higher plants employ an inorganic carbon (Ci) concentrating mechanism (CCM) to increase CO_2_ availability to Rubisco. Operation of the CCM should enhance the activity of the Calvin cycle, which could act as an electron sink for electrons generated by photosynthesis, and lower the redox status of photosynthetic electron transport chains. In this study, a hypothesis that microalgal cells with fully operating CCM are less likely to be photodamaged was tested by comparing a *Chlorella* mutant with its wild type (WT). The mutant acquired by screening gamma-ray-induced mutant libraries of *Chlorella* sp. ArM0029B exhibited constitutively active CCM (CAC) even in the presence of additional Ci sources under mixotrophic growth conditions. In comparison to the WT alga, the mutant named to constitutively active CCM1 (CAC1) showed more transcript levels for genes coding proteins related to CCM such as Ci transporters and carbonic anhydrases (CA), and greater levels of intracellular Ci content and CA activity regardless of whether growth is limited by light or not. Under photoinhibitory conditions, CAC1 mutant showed faster growth than WT cells with more PSII reaction center core component D1 protein (encoded by *psbA*), higher photochemical efficiency as estimated by the chlorophyll fluorescence parameter (Fv/Fm), and fewer reactive oxygen species (ROS). Interestingly, high light (HL)-induced increase in ROS contents in WT cells was significantly inhibited by bicarbonate supplementation. It is concluded that constitutive operation of CCM endows *Chlorella* cells with resistance to HL partly by reducing the endogenous generation of ROS. These results will provide useful information on the interaction between CCM expression, ROS production, and photodamage in *Chlorella* and related microalgae.

## Introduction

Light is essential for photosynthesis, but excess light can be damaging as it can increase the formation of reactive oxygen species (ROS) (Aro et al., 1993). These include singlet oxygen (^1^O_2_), which is produced at the reaction center of photosystem II (PSII; Vass, 2011); superoxide ($O_{2}^{–}$); and hydrogen peroxide (H_2_O_2_). The latter is generated during the Mehler-ascorbate peroxidase (MAP) cycle (Nishiyama et al., 2004; Roberty et al., 2014). ROS can directly damage PSII or inhibit its repair (Murata et al., 2007; Vass and Cser, 2009). The compounds can also result in lipid peroxidation, site-specific amino acid modifications, and mutations (Lesser, 2006). In addition to these destructive effects, ROS also plays a role as signaling molecules in various biochemical and physiological responses leading to improving stress tolerance or causing programmed cell death (Bechtold et al., 2008; Lemoine and Schoefs, 2010; Foyer and Shigeoka, 2011).

Photosynthetic organisms have several protective mechanisms against ROS-induced damage. All photosynthetic organisms perform non-photochemical quenching, which dissipates absorbed excess light energy as heat, reducing the energy reaching the PSII reaction center (Muller et al., 2001). The size of the chlorophyll-containing light-harvesting complex is regulated by the intensity of light in the environment: when light it excessive, the number of light-harvesting antenna molecules decreases, reducing the amount of light absorbed (Smith et al., 1990; Melis, 1991). The D1 protein in the reaction center of PSII is particularly susceptible to light-induced oxidative damage. PSII has efficient and dynamically regulated machinery that selectively degrades photodamaged D1 and replaces it with new D1 protein (Ohad et al., 1984; Aro et al., 1993). Photorespiration plays an important role in PSII protection against ROS (Kozaki and Takeba, 1996; Baroli and Melis, 1998; Wang et al., 2015). Recently, it was demonstrated that the addition of bicarbonate to the culture medium of a microalga alleviated oxygen stress via increase in the ratio of dissolved CO_2_ to dissolved O_2_, that would lead to lowering ROS generation (Peng et al., 2017).

Aquatic organisms must acquire the CO_2_ that they need from the surrounding water, in which it diffuses 10,000 times more slowly than it does in air. These organisms have overcome this limitation by evolving an inorganic carbon (Ci) concentrating mechanism (CCM) to increase Ci in the cell. The CCM improves the photosynthetic performance of the organism by increasing the CO_2_ concentration in the vicinity of Rubisco. This simultaneously enhances carbon fixation and suppresses photorespiration (Wang et al., 2015). The CCM contains an Ci transporter for CO_2_ and ${\mathrm{HCO}}_{3}^{–}$, a pyrenoid that serves as a barrier to CO_2_ leakage, and a carbonic anhydrase (CA) that carries out interconversion of CO_2_ and ${\mathrm{HCO}}_{3}^{–}$ (Wang et al., 2011). The CCM is induced when CO_2_ is limiting and is mainly regulated by Ci concentration (Giordano et al., 2005).

The CCM of microalgae is induced not only by limiting CO_2_ but by high light (HL) (Wang et al., 2011). Expression of several CCM genes in cyanobacteria, *Chlamydomonas*, and diatoms was induced by the combination of low CO_2_ and HL conditions (Hihara et al., 2001; Huang et al., 2002; Im and Grossman, 2002; Heydarizadeh et al., 2017), but not by the combination of HL and high-CO_2_ conditions. It may be, then, that the HL signal is insufficient for the induction of CCM genes in microalgae (McGinn et al., 2003; Woodger et al., 2003).

The CO_2_ fixed by Rubisco in the Calvin cycle is the final electron acceptor of photosynthetic electron flow, and the availability of CO_2_ affects the amount of photodamage. This was demonstrated in *Chlamydomonas reinhardtii* (Baroli and Melis, 1998) and *Dunaliella salina* (Fischer et al., 2006) grown under HL conditions. As CO_2_ was depleted in these algae, the rate of photodamage increased.

It has been suggested that activation of CCMs could reduce photodamage by increasing the CO_2_ levels in cells. If this is the case, then, the operation of the CCM that enhances Calvin cycle activity could act as a sink for the electrons generated by photosynthesis, and hence lower the redox status of photosynthetic electron transport chains. Under these conditions, cells with fully operating CCMs would be less likely to be damaged by light. This hypothesis was tested using a mutant of *Chlorella* sp. ArM0029B, a species from the arctic (Ahn et al., 2012). The mutant named to constitutively active CCM1 (CAC1) has constitutive expression of CCM and HL tolerance under mixotrophic growth conditions. The physiological and biochemical differences between the wild-type (WT) alga and CAC1 grown at low and HL conditions were characterized and compared.

## Materials and Methods

### Mutant Isolation and Culture Conditions

Cells of *Chlorella* sp. ArM0029B were cultured at 25°C in Tris–acetate–phosphate (TAP) medium (Harris, 1989). Mutants of this species were generated by irradiating cells with 300 Gy (100 Gy per hour) in a ^60^Co gamma irradiator (150 TBq Capacity; ACEL, Nordion, Ottawa, ON, Canada) at the Korean Atomic Energy Research Institute. A mutant tolerant to HL intensity was isolated from the mutant library by growing cultures under intermediate light intensity (300–350 μmol m^-2^ s^-1^). The mutant was named to “CAC1”. Cultures were grown under low [referred to as low light (LL); 50–80 μmol m^-2^ s^-1^] or HL (650–800 μmol m^-2^ s^-1^) intensities. Growth rate was assessed by counting cells under a microscope after 0.5 × 10^6^ cells ml^-1^ was inoculated in 50 ml culture.

### Identification of CCM Genes in *Chlorella* sp. ArM0029B

Carbon concentrating mechanism-related genes were identified from the genome information of *Chlorella* sp. ArM0029B as part of genome project (funded by Advanced Biomass R&D Center). A Basic Local Alignment Search Tool (BLAST) search was performed, using CCM proteins of *C. reinhardtii* as bait, to identify homologs of CCM proteins in *Chlorella.*

### Western Blot

Total proteins were extracted from WT and mutant cells according to the method of Minko et al. (1999). Total protein (1 × 10^7^ cells) was separated in a 15% (w/v) polyacrylamide gel (Laemmli, 1970) and transferred onto an Immobilon-P membrane (Millipore, Billerica, MA, United States). Rabbit anti-AtpB, LhcB2, PsaC, and PsbA polyclonal antibodies (Agrisera, Vännä, Sweden) were used to detect specific proteins. Immunodetection was performed using chemiluminescence (ECL; Amersham, Arlington Heights, IL, United States) according to the manufacturer’s instructions. Band densities were measured using ImageJ software (NIH, Bethesda, MD, United States); western blots band densities were normalized to total proteins stained with Ponceau S.

### RNA Analysis

Total RNA was extracted from WT and mutant cells (approximately 1 × 10^8^ cells) using TRIzol reagent (Invitrogen, Carlsbad, CA, United States) according to the manufacturer’s instructions. For northern blot analysis, 15 μg of total RNA was separated by agarose gel electrophoresis and blotted onto a Hybond-N nylon membrane (Amersham Biosciences, Marlborough, MA, United States). DNA fragments that had been amplified with gene-specific primers were used as probes (**Supplementary Table S1**). After hybridization with a ^32^P-labeled probe, the membrane was washed and exposed to a phospho-imaging plate (Fujifilm, Tokyo, Japan) for 1–3 days. The hybridization signals were detected with Bio-Imaging Analyzer BAS-1800II (Fujifilm, Tokyo, Japan). For semi-quantitative PCR analysis, reverse transcription (RT) was carried out using 2 μg of total RNA with oligo-dT, 200 U of murine moloney leukemia virus (M-MLV) reverse transcriptase (Promega, Madison, WI, United States), 500 μM of each dNTP, and 20 U of ribonuclease inhibitor. Semi-quantitative RT-PCR analysis was performed with 25–35 cycles using each gene-specific primer (**Supplementary Table S1**). The expression of the small subunit 3 of the Rubisco gene (*rbcS3*; MG669272) was used as a loading control. Band densities were measured using ImageJ software (NIH, Bethesda, MD, United States); for northern blots and semi-quantitative RT-PCR, band densities were normalized to loading control.

### Measurements of Intracellular Ci, Fluorescence Parameter (Fv/Fm), and Photosynthetic O_2_ Evolution

Intracellular Ci levels were measured according to Park et al. (1999), in which oxygen evolution is correlated with intracellular Ci, with some modifications. The cells (3 × 10^8^ cells) were washed three times with CO_2_-free 20 mM MES. The amount of oxygen generated by the cell was measured with an Oxylab oxygen electrode (OXYL1; Hansatech, King’s Lynn, United Kingdom). The maximal photochemical efficiency as estimated with the chlorophyll fluorescence parameter Fv/Fm determined according to Kitajima and Butler (1975) with a Handy Plant Efficiency Analyzer (Hansatech, King’s Lynn, United Kingdom). Light response curves for photosynthetic O_2_ evolution rate were measured with an Oxylab oxygen electrode (OXYL1; Hansatech, King’s Lynn, United Kingdom) at 0, 250, 500, 750, and 1,000 μmol photons m^-2^ s^-1^. Each light intensity was applied for 5 min. Chlorophyll contents were determined as previously described (Porra et al., 1989).

### Determination of Carbonic Anhydrase Activity

Carbonic anhydrase activity was determined by the potentiometric method of Wilbur and Anderson (1948) with some modifications. Cultures with 10^8^ cells were centrifuged at 3,000 rpm for 5 min and washed twice with 10 mM Tris buffer (pH 8.3) containing 1 mM dithiothreitol (DTT) and 1 mM EDTA. For detection of extracellular CA activity, the integrity of the cells in the pellet was confirmed by viewing them under the microscope. They were resuspended in the same Tris buffer and enzymatic activity was measured immediately. For detection of total CA activity, the pelleted cells were ground with a tissue lyser (QIAGEN, Hilden, Germany) in liquid nitrogen to a fine powder. Ground tissue was resuspended in the same Tris buffer and the enzymatic activity of the total extract was measured.

### *In Vivo* Detection of ROS

Production of ROS was detected by the method of Halliwell and Gutteridge (1999) with some modifications. Cell suspensions containing 1 × 10^7^ cells in 2 ml of medium supplemented with 5 μM 2′,7′-dichlorofluorescein-diacetate (DCF-DA) at 25°C were incubated in the dark for 1 h with shaking. The fluorescence of the samples was measured with a spectrofluorometer (Model LS55; PerkinElmer, Norwalk, CT, United States) at room temperature, with an excitation wavelength of 485 nm and an emission band between 500 and 600 nm. The fluorescence intensity at 520 nm (F520) was used to determine the relative ROS production.

### Statistical Analysis

For statistical analysis, SPSS software (version 25; IBM, Armonk, NY, United States) was used. The treatment effects were evaluated by a Tukey’s test (*p* < 0.01).

## Results

### High-Light-Tolerant Mutant Exhibited Constitutively Active CCM Under Mixotrophy

**FIGURE 1:**
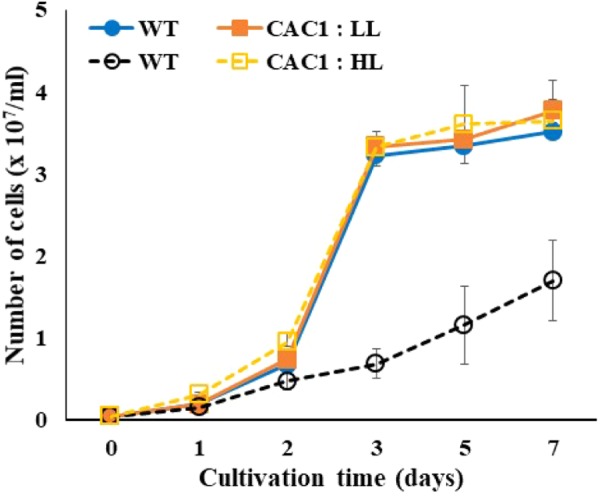
Growth of wild-type (WT) and CAC1-mutant cells under photosynthesis limiting low light (LL, 50–80 μmol m^-2^ s^-1^) and saturating, high light (HL, 650–800 μmol m^-2^ s^-1^) intensities for 7 days. Data are the means ± SE (*n* = 3).

Photosynthetic O_2_ evolution rates of *Chlorella* WT cells grown under LL or HL intensities revealed saturation behavior above 500 μmol m^-2^ s^-1^ (**Supplementary Figure S1**). In the present study, intermediate light condition (300–350 μmol m^-2^ s^-1^) was chosen for screening mutants from the mutant libraries by incubating them on agar plates. Limiting light intensity would be too low to induce photoinhibition of photosynthesis, while excess light would be too high, lowering the chance of algal cells survived. Out of about 3,000 random mutants screened, a fast-growing, HL-tolerant mutant was isolated from the intermediate light condition. This HL resistance was also observed in liquid growth conditions. Growth of this mutant and the WT grown in liquid media were similar at LL, but the mutant grew rapidly in HL where WT growth were severely inhibited (**Figure 1**).

**FIGURE 2:**
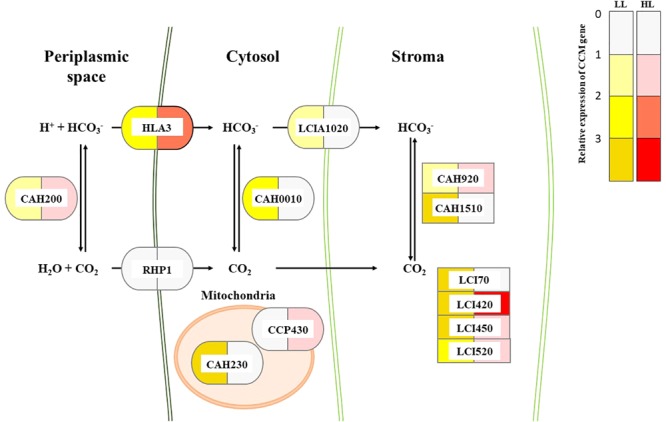
Proposed composition of CCM and relative transcript changes of CCM genes in WT and CAC1 of *Chlorella* sp. ArM0029B. Color codes indicate relative transcripts level of CCM genes in CAC1 mutants compared to WT grown under LL (50–80 μmol m^-2^ s^-1^) or HL (650–800 μmol m^-2^ s^-1^) conditions. Localization of each proteins was predicted by subcellular localization prediction tools including TargetP v1.1 (Emanuelsson et al., 2007), ChloroP v1.1 (Emanuelsson et al., 1999), SignalP v4.1 (Emanuelsson et al., 2007), SMART v8 (Letunic et al., 2014), and TMHMM v2.0 (Krogh et al., 2001). Ci transporter, CCP430 (MG669265); bicarbonate transporters, HLA3 (MG669266) and LCIA1020 (MG669271); CO_2_ channel, RHP1 (MG669273); low CO_2_ inducible proteins, LCI70 (MG669267), LCI420 (MG669268), LCI450 (MG669269), and LCI520 (MG669270); carbonic anhydrases, CAH200 (MG669261), CAH230 (MG669262), CAH0010 (MG669260), CAH920 (MG669263), and CAH1510 (MG669264). GenBank accession numbers of each genes are in parentheses.

**FIGURE 3:**
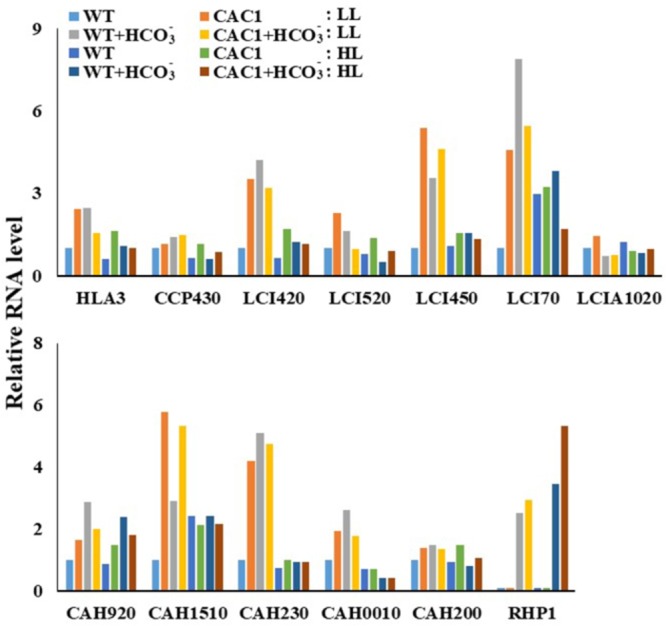
Transcript levels of CCM genes in WT and CAC1-mutant grown under photosynthesis limiting LL (50–80 μmol m^-2^ s^-1^) and saturating, HL (650–800 μmol m^-2^ s^-1^) intensities for 3 days. The Northern blot and RT-PCR bands were quantified from **Supplementary Figure S2** and shown as relative abundance normalized to the WT expression level, which is considered as 1.0.

To correlate CCM machinery with HL tolerance of the mutant, transcript levels of CCM genes, CA activity, and intracellular Ci content were assessed. Genome of *Chlorella* sp. ArM0029B contains CCM genes such as : two bicarbonate transporter genes [HLA3 and LCIA1020; homologs for HL-induced gene 3 (HLA3) and low CO_2_-induced gene A (LCIA) of *C. reinhardtii*], one CO_2_ channel gene [RHP1; homolog for rhesus-like proteins 1 and 2 (RHP1/2) of *C. reinhardtii*], one Ci transporter gene [CCP430; homolog for low-CO_2_-inducible chloroplast envelope proteins 1 and 2 (CCP1/2) of *C. reinhardtii*], four low-CO_2_-inducible genes [LCI420, LCI450, LCI70, and LCI520; homologs for low CO_2_-induced gene B, C, D, and E (LCIB, LCIC, LCID, and LCIE) of *C. reinhardtii*], and five CA genes [CAH230, CAH200, CAH920, CAH1510, and CAH0010; homologs of CA 1, 2, 3, 5, 7, and 9 (CAH1, CAH2, CAH3, CAH5, CAH7, and CAH9) of *C. reinhardtii*]. More information is included in **Figure 2**. Transcript levels of these genes assessed by Northern and RT-PCR analyses demonstrated that most of *CCM* genes were constitutively and highly expressed in mutant, relative to the WT (**Figure 3** and **Supplementary Figure S2**). In the WT alga, expression of genes encoding Ci transporters and CAs in the HL was lower (0.61–0.96-fold) than in LL (**Figure 3**), while in HL, there were 1.24–2.99-fold more transcripts of *LCI70, LCIA1020*, and *CAH1510* than were present in LL. Most of CCM genes, specifically *HLA3, CCP430, LCI420, LCI520, LCI450, LCI70, CAH920, CAH1510, CAH230, CAH0010*, and *RHP1* were 1.42–7.87-fold highly expressed when cultures were supplemented with bicarbonate, compared to bicarbonate-free cultures, although expression of *LCIA1020* and *CAH200* was decreased (0.67–0.71-fold) and 1.51–0.67-fold increased, respectively, in bicarbonate. Expression of CCM genes was either unchanged or slightly lower (0.46–0.91-fold) when cultures were supplemented with high CO_2_ (**Supplementary Figure S3**).

There was more expression of the CCM genes in the mutant than in the WT under both light regimes. Especially, the expression of genes encoding the HLA3 homolog and the LCIB/C homologs was 1.15–5.39-fold more increased in the mutant compared to the WT. In contrast, expression of *LCI520, LCI450, LCI70*, and *LCIA1020* in the mutant was slightly lower (0.29–0.7-fold) in HL than in LL, but the expression was still higher (1.1–1.75-fold) except for *LCIA1020* in the mutant than in the WT. Similarly, while the expression of *HLA3, LCI420*, and *LCI520* was less (0.42–0.91-fold) when the mutant was supplemented with bicarbonate, these were also still 1.17–1.69-fold greater in the mutant than in the WT. Expression of *RHP1* in algae was detected only in when bicarbonate was supplied. Expression of this gene was similar in both algae under LL, but was 1.55-fold higher in the mutant than in the WT under HL (**Figure 3**).

**FIGURE 4:**
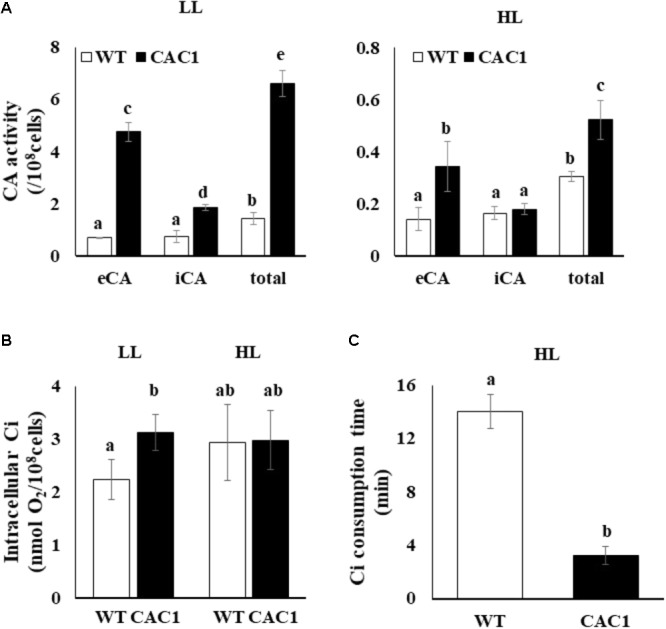
Carbonic anhydrase (CA) activity **(A)**, intracellular inorganic C (Ci) content **(B)**, and intracellular Ci consumption time **(C)** in WT and CAC1 mutant grown under photosynthesis limiting LL (50–80 μmol m^-2^ s^-1^) and saturating, HL (650–800 μmol m^-2^ s^-1^) intensities for 3 days. eCA, extracellular CA activity; iCA, intracellular CA activity; total, eCA activity + iCA activity; data are means ± SE (*n* = 3). Means denoted by different letters indicated significant difference at *p* < 0.01 according to Tukey’s test.

Carbon concentrating mechanism machinery is massively under the transcriptional control (Brueggeman et al., 2012). Thus, differential expression of CAHs between the WT and the mutant as described above should be manifested as changes in CA activities. As expected, CA activity in both algae was less in HL than in LL, but the CA activity in the mutant was much greater than in the WT (**Figure 4A**). Compared to the WT alga, under LL, the total CA activity of the mutant was 4.5 times greater; extracellular activity was seven times greater, and intracellular CA activity was 2.5 times greater. Compared to the WT, under HL, the total CA activity of mutant was 1.7 times greater, extracellular CA activity was 2.4 times greater, while intracellular CA activity was similar in the two algae.

Cells with a CAC would contain high content of intracellular Ci. Indeed, in LL, the intracellular Ci content in the mutant was 1.4 times greater than that of the WT (**Figure 4B**). However, intracellular Ci in the WT and the mutant was comparable under HL (**Figure 4B**). Interestingly, the mutant consumed the intracellular Ci 4.2-fold faster than that in WT (**Figure 4C**). Overall, the mutant isolated under the intermediate light showed a phenotype with CAC and hence was named as CAC1.

### Photosynthetic Apparatus, ROS, and Growth Performance

**FIGURE 5:**
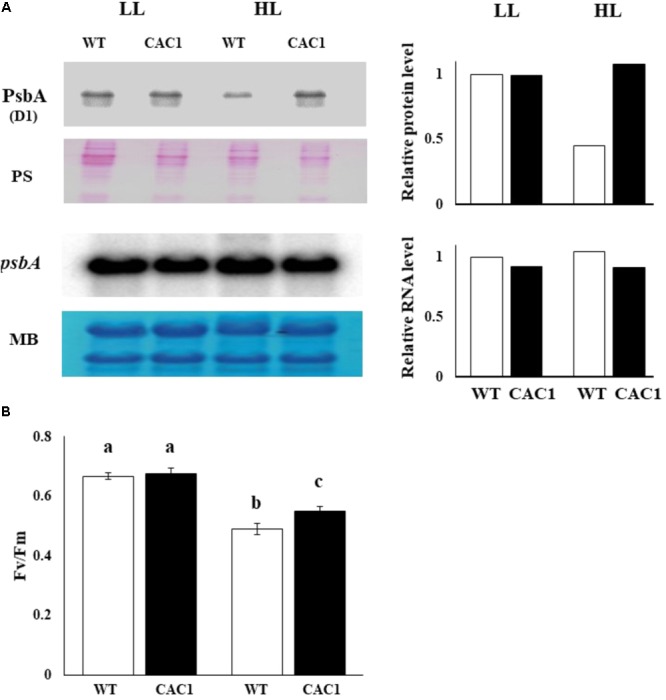
Western and northern analyses of PSII core protein PsbA **(A)** and maximal photochemical efficiency of PSII (Fv/Fm) **(B)** of WT and CAC1 mutant grown under photosynthesis limiting LL (50–80 μmol m^-2^ s^-1^) and saturating, HL (650–800 μmol m^-2^ s^-1^) intensities for 3 days. The Western blot bands were quantified and shown as relative abundance normalized to D1 (PsbA) accumulation level in the WT, which is considered as 1.0. Data are means ± SE (*n* = 4). MB, methylene blue staining; PS, Ponceau S-staining. Means denoted by different letters indicated significant difference at *p* < 0.01 according to Tukey’s test.

If CCM have a role for lowering the extent of PSII photodamage, the primary target of photoinhibition, then CAC1 with robust CCM grow faster than WT cells under HL condition. First, the extent of photodamage in the WT and mutant algae grown under HL was compared by assessing the amount of D1 protein, PSII photochemical efficiency, photosynthetic O_2_ evolution rate, and cell growth curves. These analyses were done in cells cultured for 3 days. Fast growing phase of the WT and CAC1 supplemented with additional bicarbonate observed between 2 and 3 days after inoculation, followed by stationary phase (**Figure 1**). Three-day old cells rather than stationary phase cells were chosen to avoid the involvement of other factors such as high-density-related nutrient deficiency and self-shading in the process of PSII photodamage and repair processes. In the WT, there was less (0.49-fold) D1 protein (PsbA) in HL than in LL, although the *psbA* expression was not changed, indicating that the WT cells were under the photoinhibitory condition. In the mutant, however, levels of the D1 protein as well as expression of *psbA* were similar in both light regimes (**Figure 5A**). Consistent with D1 protein levels, maximal photochemical efficiency of PSII estimated as a fluorescence parameter, Fv/Fm, and maximal photosynthetic O_2_ evolution rate of HL adapted WT cells was 73 and 36% of the CAC1, respectively (**Figure 5B** and **Supplementary Figure S1**). Taken together, these data strongly indicate that CAC1 are more resistant than the WT to HL stress.

**FIGURE 6:**
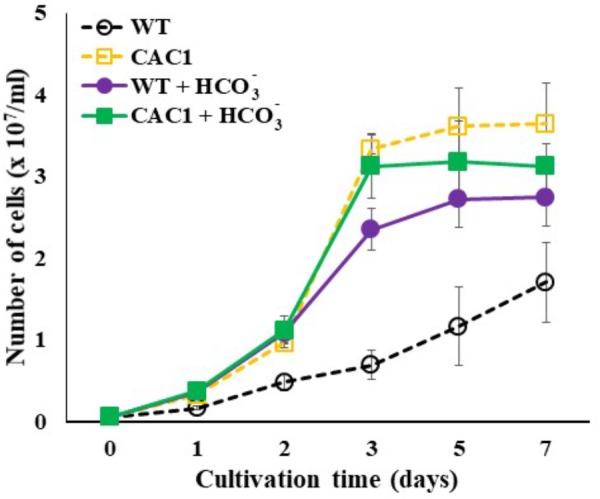
Growth of WT and CAC1 mutant supplemented with Ci source (10 mM NaHCO_3_) under HL (650–800 μmol m^-2^ s^-1^) intensities for 7 days. Data are means ± SE (*n* = 3).

If HL resistance observed in CAC1 mutant is related to enhanced Ci content, then bicarbonate or gaseous CO_2_ supplies would restore growth of the WT cells comparable to CAC1 under HL. This view was tested by assessing cell growth supplied with either bicarbonate (10 mM) or high CO_2_ (2%) under intermediate light. A spot growth test (visualization of growth on agar medium) demonstrated that growth of both the WT and CAC1 was slightly increased in high CO_2_ compared to the non-supplemented controls, but CAC1 still grew faster than the WT (**Supplementary Figure S4**). However, bicarbonate supplementation markedly increased growth of the WT cells, being similar to that of CAC1 (**Supplementary Figure S4**). This apparent restoration was also apparent in liquid culture under HL condition (**Figure 6**), supporting the view that CCM has a photoprotective role against HL stress.

**FIGURE 7:**
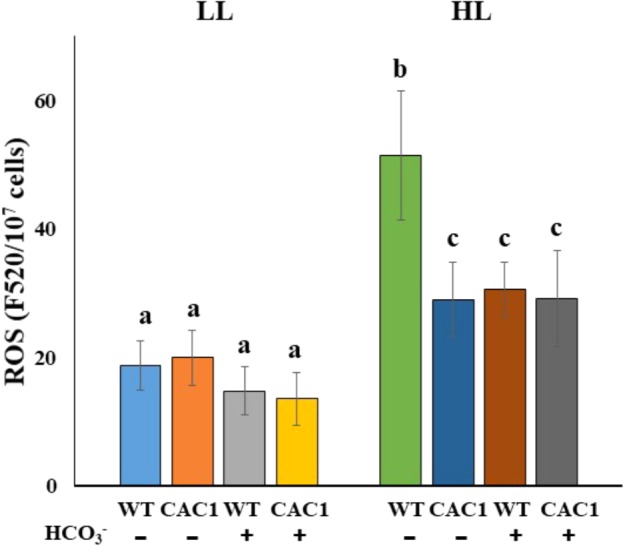
ROS contents in WT and CAC1 mutant grown under photosynthesis limiting LL (50–80 μmol m^-2^ s^-1^) and saturating, HL (650–800 μmol m^-2^ s^-1^) intensities for 3 days. Data are means ± SE (*n* = 4). Means denoted by different letters indicated significant difference at *p* < 0.01 according to Tukey’s test.

Finally, a positive role of CCM against photodamage was predicted to be that, first, CAC1 cells would have lowered ROS contents and, second, enhanced Ci levels alleviate ROS contents. As predicted, in HL CAC1 contained less (0.56-fold) ROS contents compared to the WT cells (**Figure 7**). In HL compared to LL, ROS content increased 2.74-fold in the WT but 1.45-fold in the mutant (**Figure 7**). Further, bicarbonate supplementation to the WT cells significantly inhibited increases in ROS contents. In HL WT cells without additional bicarbonate contained 1.68-fold higher ROS than those supplemented with bicarbonate (**Figure 7**).

## Discussion

In photosynthetic organisms, including algae, HL leads to the generation of ROS, which damages the photosynthetic apparatus and impairs growth (Murata et al., 2007; Vass and Cser, 2009). Enhanced Ci contents would lower ROS generation via enhanced utilization of reducing power and ATP generated during light-dependent reaction of photosynthesis. In the present study, this view was supported by characterization of the CAC1 mutant of an arctic species of *Chlorella* sp. ArM29B.

Constitutively active CCM1 is tolerant to light intensity above 150 μmol m^-2^ s^-1^ where the growth of the WT begins to be compromised (data not shown). In HL-grown WT, cell growth was severely reduced due to PSII photodamage as revealed by lowered D1 protein content, maximal photochemical efficiency of PSII (estimated by Fv/Fm parameter), and maximal photosynthesis rate. In contrast, these parameters in the CAC1 mutant were less affected in the same light treatment, indicating that CAC1 mutant is more resistant to photodamage than WT.

Reactive oxygen species can induce photodamage, which reduces photosynthesis and growth. ROS content increased drastically in HL in the WT, but there was less effect in the mutant (**Figure 7**). The addition of bicarbonate to the WT cells in HL reduced the content of ROS to levels comparable to those in CAC1. As expected, bicarbonate addition to the WT culture in HL increased the accumulation of the D1 protein (**Supplementary Figure S5**). It appears that the bicarbonate enabled the WT alga to “mimic” the performance of the mutant phenotypically. The WT cells appear to possess protective mechanisms against HL, but ROS may accumulate above the cells’ capacity for protection, and directly damage the cells’ photosystems. It may be, then, that the cellular damage inflicted by ROS could be alleviated by the addition of effective electron acceptors. Of note here is that the CO_2_ fixed by Rubisco in the Calvin cycle is the final electron acceptor in photosynthetic electron flow (Baroli and Melis, 1998). More Ci was available in CAC1 due to its constitutive CCM expression (**Figure 4B**); harmful excess electrons may have been consumed by the Calvin cycle in CAC1 and could not generate excess ROS and cause photodamage. Furthermore, the bicarbonate effect observed may assist electron transfer to PSII by a light-induced primary charge separation and subsequent water oxidation (Wydrzynski and Satoh, 2005; Shevela et al., 2012); this would be achieved by increased consumption of bicarbonate in the thylakoid lumen of either the CCM-activated-CAC1 cell or the bicarbonate-supplied WT cell in HL.

**FIGURE 8:**
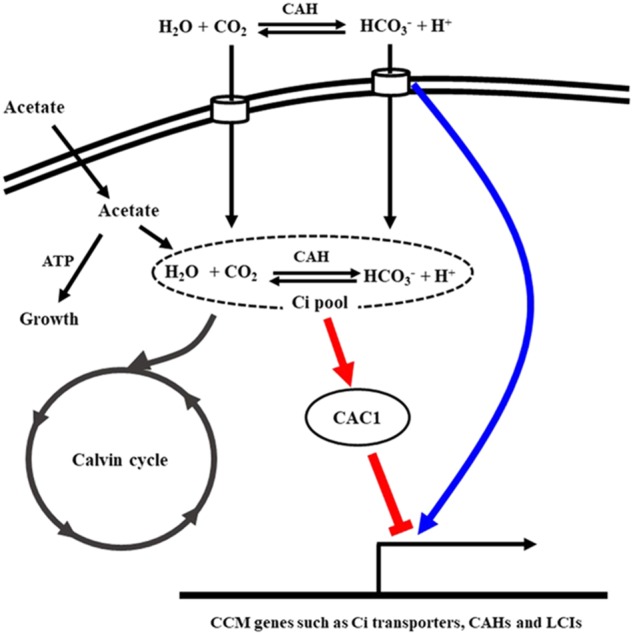
A proposed model for dual Ci signaling pathways in *Chlorella* sp. ArM0029B. In *Chlorella* sp. ArM0029B, expression of CCM genes is suppressed by high concentration of intracellular Ci mostly derived from the acetate assimilatory pathway. Under this photoheterotrophic growth conditions, bicarbonate sensing and signaling pathway (blue line) are not operating. When high concentration of ${\mathrm{HCO}}_{3}^{–}$ is exogenously provided, this bicarbonate pathway becomes active. Constitutive operation of CCM machinery observed in CAC1 mutant implicates that CAC1 suppresses Ci signaling pathway to the CCM genes (red line). Failure of high exogenous CO_2_ in CCM activation implicates the presence of membranous CO_2_ transporter that allows CO_2_ entry fast enough not to be hydrated by external CAHs. Thus, dual signaling pathways for external bicarbonate and internal Ci to the CCM apparatus are probably present in *Chlorella* sp. ArM0029B. CAH, carbonic anhydrase; Ci, inorganic carbon; LCI, low CO_2_ inducible protein.

Carbon concentrating mechanism in most algae and cyanobacteria is down regulated in a high inorganic or organic carbon environment (Moroney et al., 1987; Fett and Coleman, 1994; Iglesias-Rodriguez and Merrett, 1997; Wang et al., 2011). In the present study, cells were cultured with acetate containing TAP medium as an organic carbon source. Thus, CCM in the WT cells may be largely down regulated due to high carbon environment created by acetate. Contrary to this expectation, the expression of many CCM genes, particularly *HLA3, CCP430, LCI420, LCI520, LCI450, LCI70, CAH920, CAH1510, CAH230, CAH0010*, and *CAH200*, was increased in the WT when bicarbonate was included in the medium even in the presence of acetate (**Figure 3**). These results suggest that a different bicarbonate-sensing machinery is present with Ci signaling pathway in this alga *Chlorella* sp. ArM0029B and/or the bicarbonate-driven-Ci signal inside or outside of cell may bypass the Ci concentration recognition system (sensor) under high organic carbon environment (**Figure 8**). Insensitivity of CCM gene expression in the WT in response to high CO_2_ (**Supplementary Figure S3**) favors the presence of two independent carbon sensors (**Figure 8**). Similarly, the cyanobacterium *Synechococcus* PCC7942 cells grown in a high Ci environment readily took up CO_2_ but not ${\mathrm{HCO}}_{3}^{–}$ (Price and Badger, 1989). Contrary to WT cells, CAC1 cells exhibited constitutive expression of CCM genes even in the presence of organic carbon source. Insensitivity of ROS production in CAC1 against exogenous bicarbonate supply under HL conditions implicates the involvement of ROS signaling cascade in CCM expression. Thus, further molecular genetic studies will reveal possible different machineries sensing bicarbonate or CO_2_ and signaling to and role of relation to ROS signaling to CCM expression.

It is concluded that CAC1 constitutively operates its CCM, which causes high levels of Ci to accumulate in its cells. Based on these results, it is proposed that operation of the CCM acts as an electron sink for the electrons generated by photosynthesis: activation of the CCM provides more CO_2_ for fixation by Rubisco, which enhances Calvin cycle activity. It would follow, then, that constitutive operation of CCM endows *Chlorella* cells with resistance to HL partly by lowering endogenous generation of ROS. Present study will provide useful information on the interaction between CCM expression, ROS production, and photodamage in *Chlorella* and related microalgae.

## Author Contributions

W-JJ designed the experiments and wrote the manuscript. KH, J-ML, S-WJ, and JV performed the experiments. Y-IP analyzed the data and commented on the manuscript. All authors contributed to writing the manuscript.

## Conflict of Interest Statement

The authors declare that the research was conducted in the absence of any commercial or financial relationships that could be construed as a potential conflict of interest.

## Supplementary Material

FIGURE S1 Light response curve of photosynthetic O_2_ evolution of wild-type (WT) and CAC1 mutant grown under photosynthesis limiting low light (LL, 50–80 μmol m^-2^ s^-1^) and saturating, high light (HL, 650–800 μmol m^-2^ s^-1^) intensities for 3 days. Data are means ± SE (*n* = 3).

FIGURE S2 Transcript levels of CCM genes in WT and CAC1 mutant grown under photosynthesis limiting LL (50–80 μmol m^-2^ s^-1^) and saturating, HL (650–800 μmol m^-2^ s^-1^) intensities for 3 days. MB, methylene blue staining.

FIGURE S3 Transcripts analyses of CCM genes WT and CAC1 mutant grown in air (low CO_2_) and high CO_2_ (2%) limiting LL (50–80 μmol m^-2^ s^-1^) for 3 days. The RT-PCR bands were quantified and shown as relative abundance normalized to the WT expression level, which is considered as 1.0.

FIGURE S4 Growth of WT and CAC1 mutant supplemented with Ci sources under limiting LL (50–80 μmol m^-2^ s^-1^) and intermediate light (IL, 350–400 μmol m^-2^ s^-1^) conditions for 4 days. Ci was supplied as 2% CO_2_ or 10 mM NaHCO_3_.

FIGURE S5 PsbA content as assessed by Western blotting in WT and CAC1 mutant grown under saturating, HL intensity (650–800 μmol m^-2^ s^-1^) with (+) or without (-) 10 mM bicarbonate supplementation for 3 days. PS, Ponceau S-staining. The Western blot bands were quantified and shown as relative abundance normalized to D1 (PsbA) accumulation level in the WT, which is considered as 1.0.

TABLE S1 Sequence of the primers used in this study.
