# Supplementary figures and images for: Elevated Inorganic Carbon Concentrating Mechanism Confers Tolerance to High Light in an Arctic Chlorella sp. ArM0029B
Source: Front Plant Sci. 2018 May 7;9:590. doi: 10.3389/fpls.2018.00590 (PMC5949578; doi:10.3389/fpls.2018.00590)

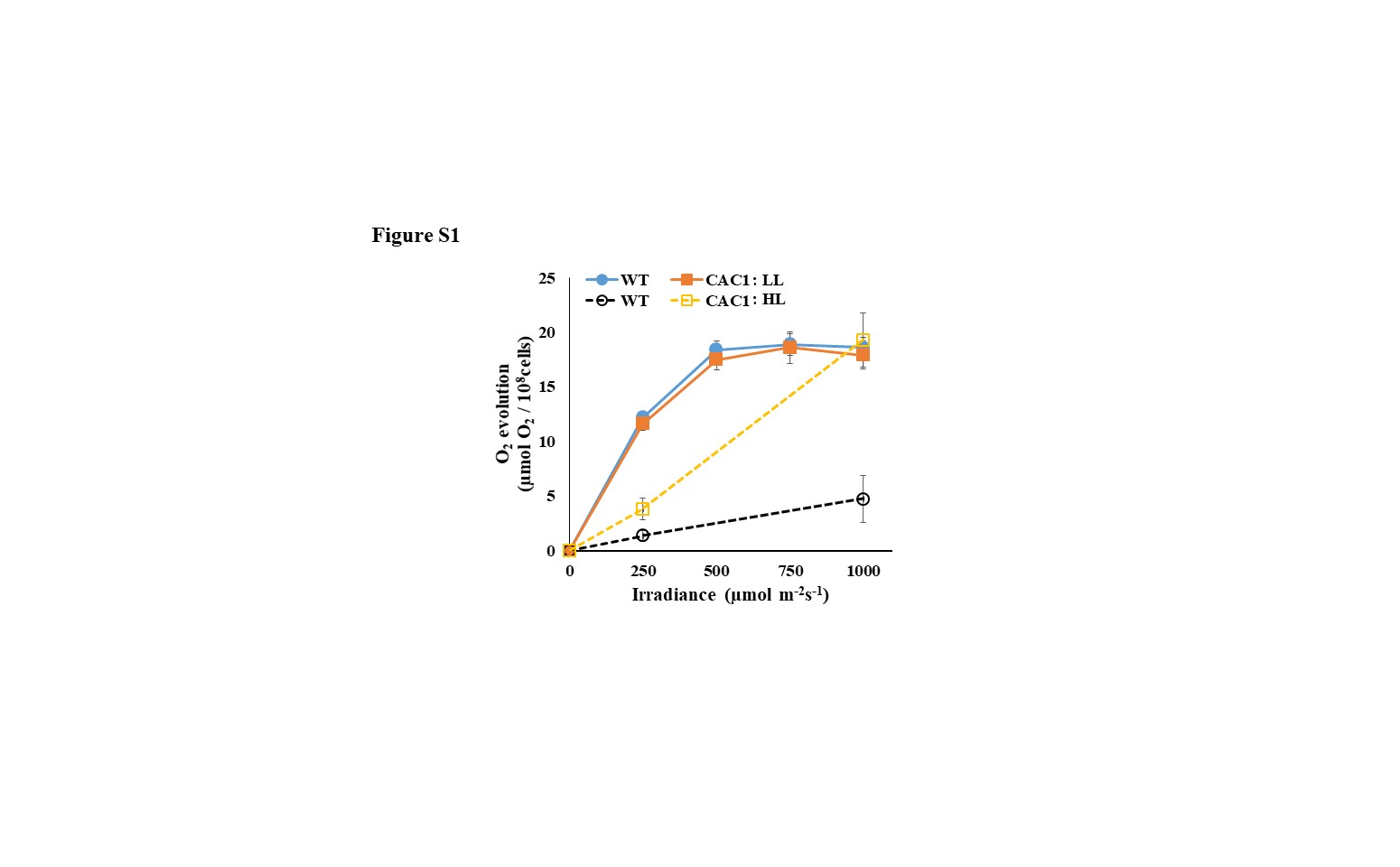

Supplement: FIGURE S1 — Light response curve of photosynthetic O2 evolution of wild-type (WT) and CAC1 mutant grown under photosynthesis limiting low light (LL, 50–80 μmol m-2 s-1) and saturating, high light (HL, 650–800 μmol m-2 s-1) intensities for 3 days. Data are means ± SE (n = 3). [file Image_1.JPEG]

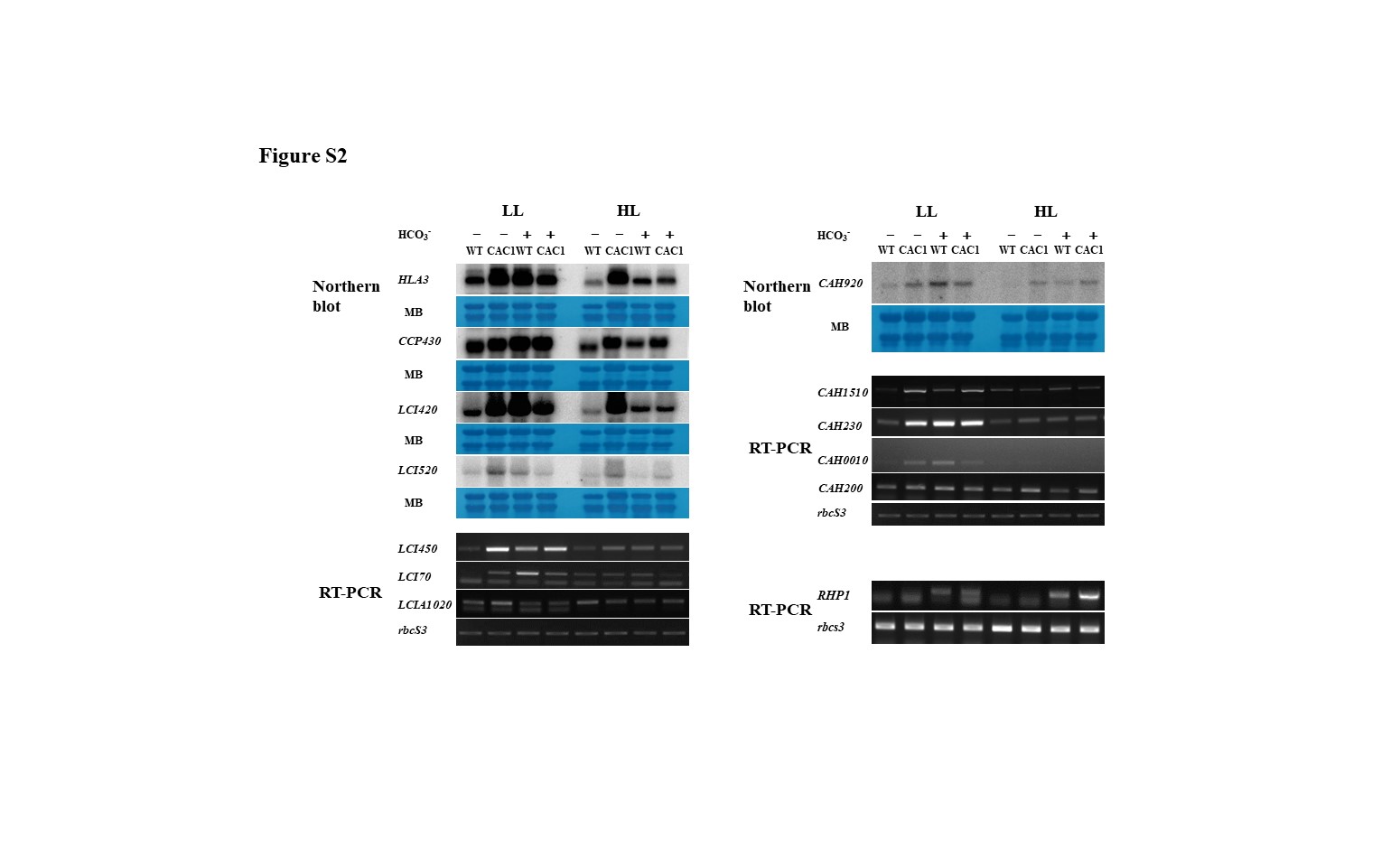

Supplement: FIGURE S2 — Transcript levels of CCM genes in WT and CAC1 mutant grown under photosynthesis limiting LL (50–80 μmol m-2 s-1) and saturating, HL (650–800 μmol m-2 s-1) intensities for 3 days. MB, methylene blue staining. [file Image_2.JPEG]

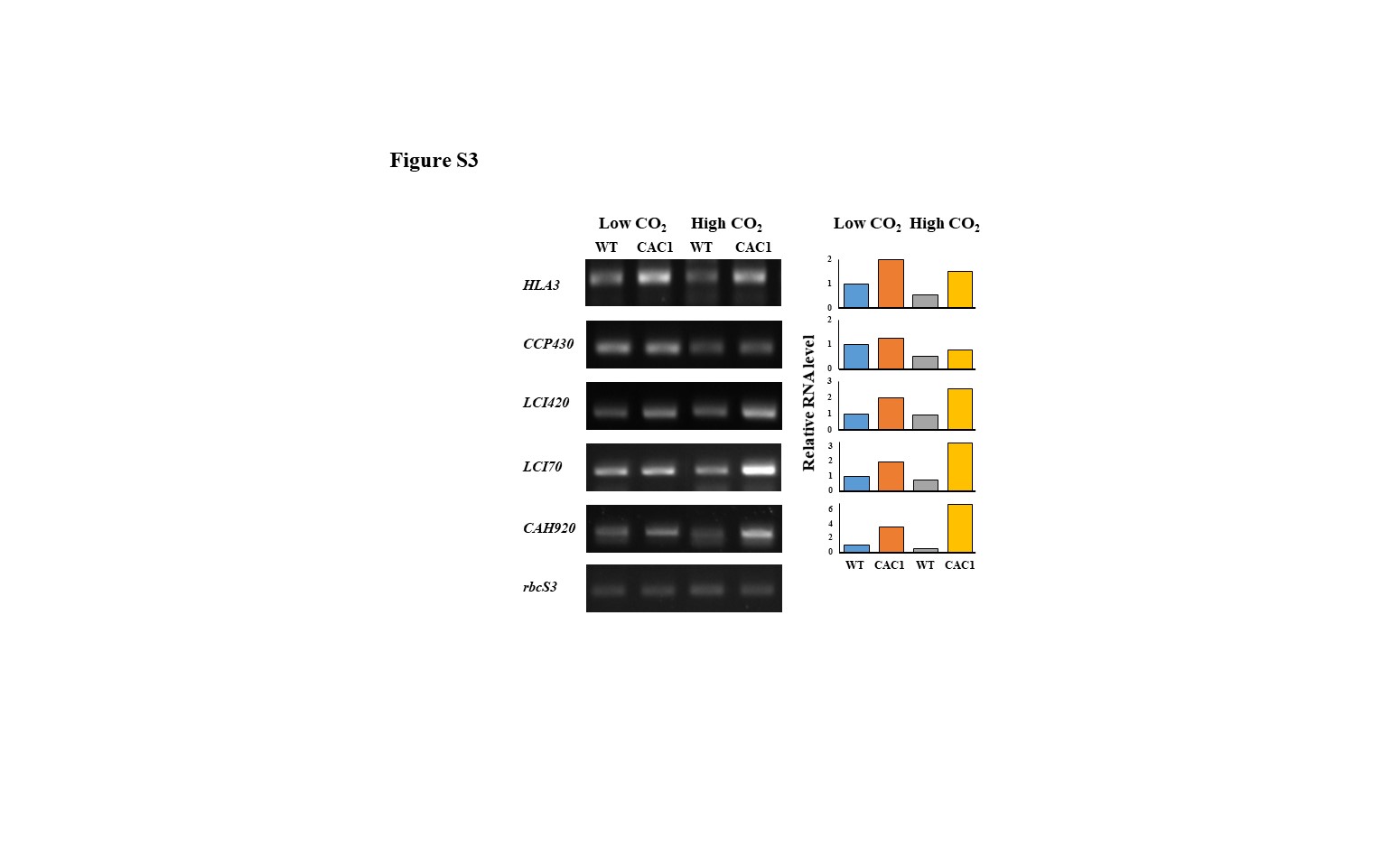

Supplement: FIGURE S3 — Transcripts analyses of CCM genes WT and CAC1 mutant grown in air (low CO2) and high CO2 (2%) limiting LL (50–80 μmol m-2 s-1) for 3 days. The RT-PCR bands were quantified and shown as relative abundance normalized to the WT expression level, which is considered as 1.0. [file Image_3.JPEG]

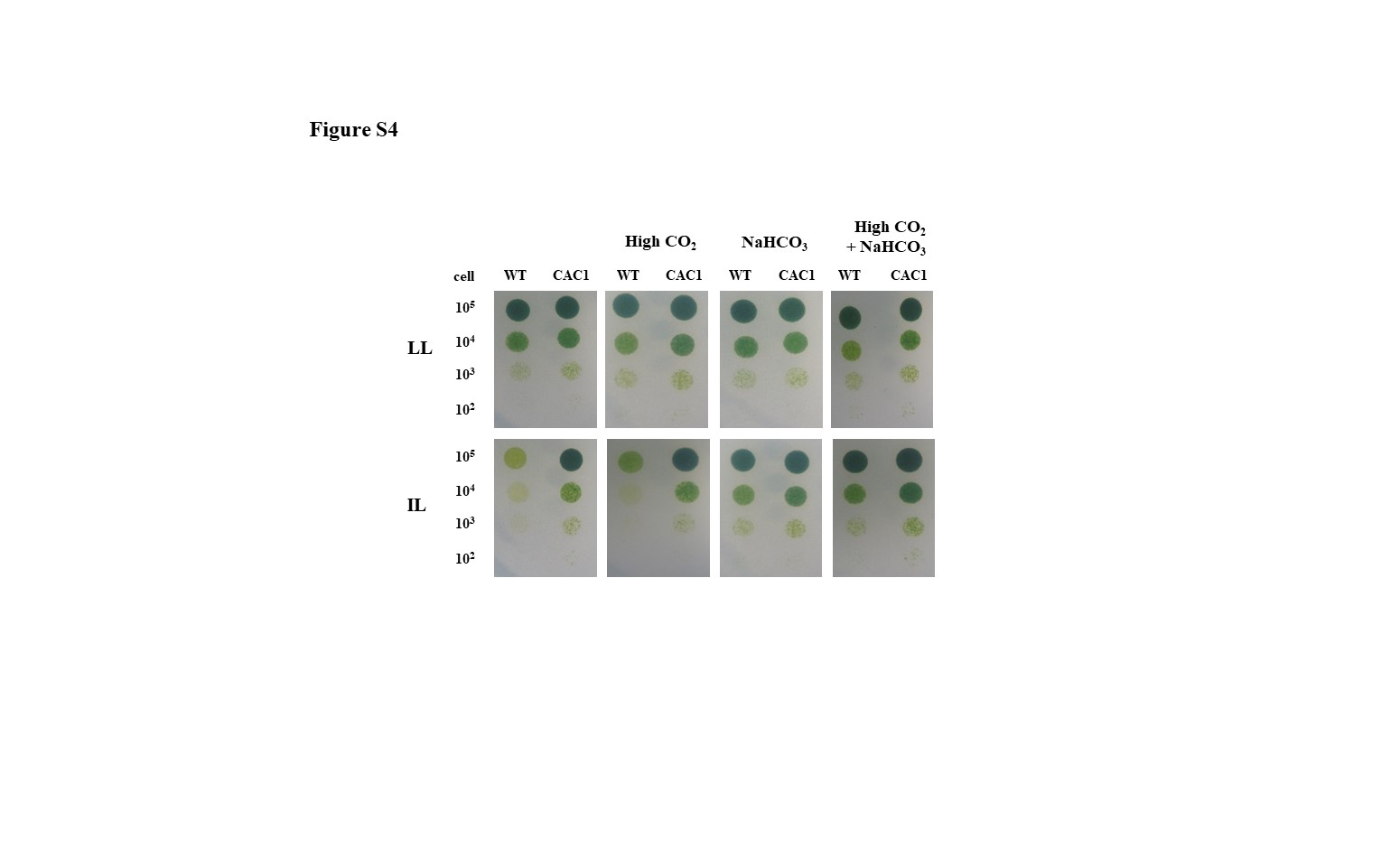

Supplement: FIGURE S4 — Growth of WT and CAC1 mutant supplemented with Ci sources under limiting LL (50–80 μmol m-2 s-1) and intermediate light (IL, 350–400 μmol m-2 s-1) conditions for 4 days. Ci was supplied as 2% CO2 or 10 mM NaHCO3. [file Image_4.JPEG]

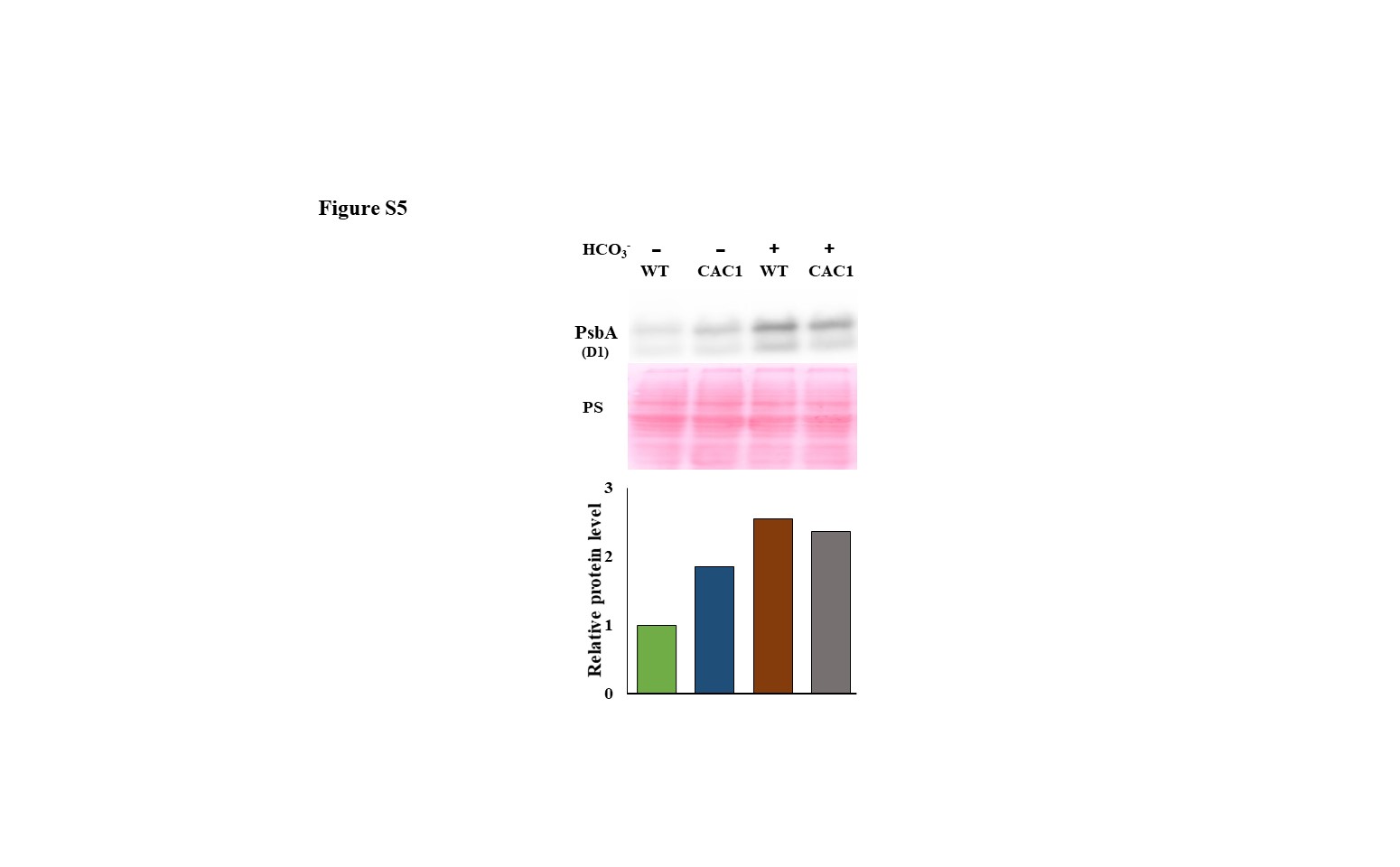

Supplement: FIGURE S5 — PsbA content as assessed by Western blotting in WT and CAC1 mutant grown under saturating, HL intensity (650–800 μmol m-2 s-1) with (+) or without (-) 10 mM bicarbonate supplementation for 3 days. PS, Ponceau S-staining. The Western blot bands were quantified and shown as relative abundance normalized to D1 (PsbA) accumulation level in the WT, which is considered as 1.0. [file Image_5.JPEG]
