# Supplementary material for: Elevated Inorganic Carbon Concentrating Mechanism Confers Tolerance to High Light in an Arctic Chlorella sp. ArM0029B
Source: Front Plant Sci. 2018 May 7;9:590. doi: 10.3389/fpls.2018.00590 (PMC5949578; doi:10.3389/fpls.2018.00590)
Supplement: TABLE S1 — Sequence of the primers used in this study. [file Table_1.DOCX]

**Supplementary Table 1. Sequence of the primers used in this study.**

| Gene | Forward primer | Reverse Primer |
| --- | --- | --- |
| HLA3 | 5'- CATGCGGTGGCCTGGACTCCA -3' | 5'- TGGTCAGAGCGGATGACGGTGTC -3' |
| CCP430 | 5'- GCTGGCGGGACCCAACAAGAC -3' | 5'- GACTGGCGCAGGCAGTCCAT -3' |
| LCI420 | 5'- GCCAGCATTGGTGGGCAGGA -3' | 5'- CTTGTCGTCCAGCTTGGGTGCATT -3' |
| LCI520 | 5'- TGCATGCCTACGACTCCAAGGATG -3' | 5'- GGCCTGCAGCTGCTTGGTGGACT -3' |
| LCI450 | 5'- CCATGGTGAACCTGTGCCGT -3' | 5'- ACCTCTCCCTTGGCATTGATTGA -3' |
| LCI70 | 5'- ATGACGAGCTGGATGGGATGT -3' | 5'- GGAGAGACAGCCAGGCTAGT -3' |
| LCIA1020 | 5'- AGCTCCTCCTCCTTTAATGCCAA -3' | 5'- AGCAGCACGATCTTCCACCA -3' |
| CAH920 | 5'- GGACCGCCTTGTTGAGTTCAATGT -3' | 5'- TGAGTGAGTTGAGGGAGGTGTT -3' |
| CAH1510 | 5'- GCCAAGCGTGTGCTGCGAGT -3' | 5'- GCATCAATGGGAGGCAGCAGGAT -3' |
| CAH230 | 5'- CCCTGGACCTACGACTACTCT -3' | 5'- GTGGAACTGGAGTGGCTTGA -3' |
| CAH0010 | 5'- GGCATGGTGCCTCTCGCAGA -3' | 5'- GCACTCTAGGGTGGCTTTGGCTAT -3' |
| CAH200 | 5'- GAGGCCGGCAAGGACTGCAA -3' | 5'- AGGGCAGTCTGCAGGGCAATC -3' |
| RHP1 | 5'- TTTTGCGTGATGAACACCGTCTTG -3' | 5'- ACAGGTAGCCGCAGGTGGAGAG -3' |
| rbcS3 | 5'- CAAGAAGACCACCGCCAT -3' | 5'- GAGCAGCTGTCGTTGCTG -3' |
